# Commercially available novel device to prevent the diffusion of droplets from subjects undergoing esophagogastroduodenoscopy: A pilot study with its prototype

**DOI:** 10.1002/deo2.36

**Published:** 2021-09-01

**Authors:** Hiroyuki Endo, Tomoyuki Koike, Noriyuki Obara, Waku Hatta, Atsushi Masamune

**Affiliations:** ^1^ Department of Gastroenterology Japan Community Health Care Organization Sendai Hospital Miyagi Japan; ^2^ Division of Gastroenterology Tohoku University Graduate School of Medicine Miyagi Japan

**Keywords:** COVID‐19, device, endoscopy, precaution, SARS‐CoV‐2

## Abstract

**Introduction:**

Under the current pandemic situation of the coronavirus disease 2019 (COVID‐19), we have newly developed a commercially available device named Endomask to prevent the diffusion of droplets from subjects undergoing esophagogastroduodenoscopy (EGD). Herein, we evaluate the efficacy and safety of the device, and also evaluate the stress of the device on the operators and the subjects of EGD.

**Methods:**

The efficacy of the device was evaluated using an experimental model that simulated the environment of EGD. The safety of the device was evaluated clinically by means of measuring the oxygen saturation and the expiratory carbonic dioxide partial pressure of subjects with our device during EGD. The stress of the device on the operability of the endoscopists and the respiration of the subjects were evaluated using questionnaires.

**Results:**

In the experiments with Endomask, the percentage of the area with simulated droplets was significantly reduced compared to that without our device (median, 0.24% vs. 6.96%, *p* = 0.009). The saturation of oxygen and the expiratory carbonic dioxide partial pressure of subjects with the device did not show significant change at any recording times. Neither the operators nor the subjects felt serious stress from examination with the device.

**Conclusions:**

Endomask could remarkably and safely prevent the diffusion of droplets without serious stress. Endomask is expected to contribute to a reduction of the infectious risk of SARS‐CoV‐2 in endoscopy units during COVID‐19 pandemic.

**Abbreviations**
COVID‐19: coronavirus disease 2019
EGD: esophagogastroduodenoscopy
PPE: personal protective equipment
SARS‐CoV‐2: severe acute respiratory syndrome coronavirus‐2

## INTRODUCTION

Under the current pandemic situation of coronavirus disease 2019 (COVID‐19), the infectious risk of severe acute respiratory syndrome coronavirus‐2 (SARS‐CoV‐2) in endoscopy units is enhanced.^1^, ^2^ In addition to the risk of contact and droplets transmission, the possibility of airborne transmission by aerosols should be considered when developing strategies against SARS‐CoV‐2 transmission.^3^, ^4^, ^5^ The Japan Gastroenterological Endoscopy Society (JGES) issued recommendations for gastrointestinal endoscopy during this pandemic situation.^6^, ^7^ Triage (risk stratification) of subjects, appropriate use of personal protective equipment (PPE) of medical staffs, careful management of subjects and the endoscopic room are recommended.

In addition to these strategies, a further solution for preventing the diffusion of droplets/aerosols from subjects is important in terms of reduction of the infectious risk of SARS‐CoV‐2 in endoscopic units. The presence of asymptomatic SARS‐CoV‐2‐infected patients^8^, ^9^, ^10^, ^11^ and the possibility of false negatives from polymerase chain reaction (PCR)^12^ suggest that even subjects triaged as low risk may be a source of SARS‐CoV‐2 transmission. Accordingly, a simple and inexpensive solution that can be adapted for all subjects is needed. The safety of the subjects and the stress for the endoscopists should be also taken into account when developing a solution. Although some devices for the prevention of the diffusion of droplets from subjects undergoing esophagogastroduodenoscopy (EGD) have been reported recently,^13^, ^14^, ^15^, ^16^, ^17^, ^18^, ^19^, ^20^, ^21^ there were no reports that evaluated the influence on the respiration of the subjects and the operability of the endoscopists.

**FIGURE 1:**
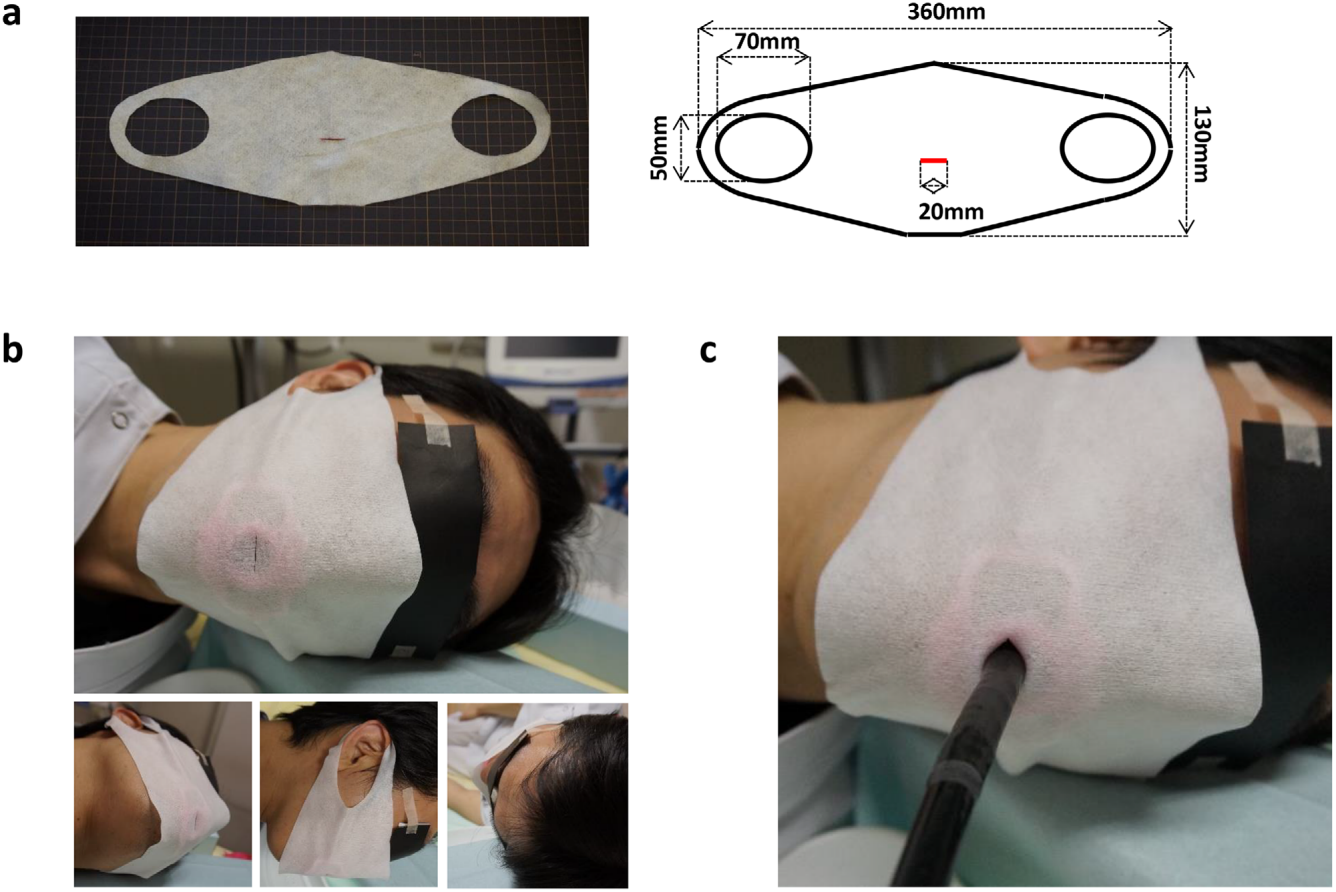
Design of Endomask and an image of a subject wearing Endomask. (a) Design of Endomask is shown. The red line is a slit for passage of the endoscope. (b) Endomask can cover most of the mouth and nose. (c) Endoscope can pass through the slit without severe interference

We previously reported a disposable self‐made device that is expected to capture droplets from subjects undergoing EGD.^22^ Although the device could be utilized even in an emergent condition lacking resources such as PPE, efforts to improvise and regard for the type of mouthpiece were necessary. Herein, we have newly developed another commercially available device named Endomask (TOP Corporation, Tokyo, Japan) to prevent the diffusion of droplets from subjects during EGD. The design of our novel device and the image of a subject with our device are shown in Figure 1. Endomask is made of a monolayer of nonwoven fabric with a slit for the passage of an endoscope. It can be utilized without regard for the type of mouthpiece. The price is 35 yen.

In this pilot study, we evaluated the efficacy and safety of Endomask using its prototype. Stress on the operability of the endoscopists and the respiration of the subjects were also evaluated.

## METOHDS

### Efficacy

**FIGURE 2:**
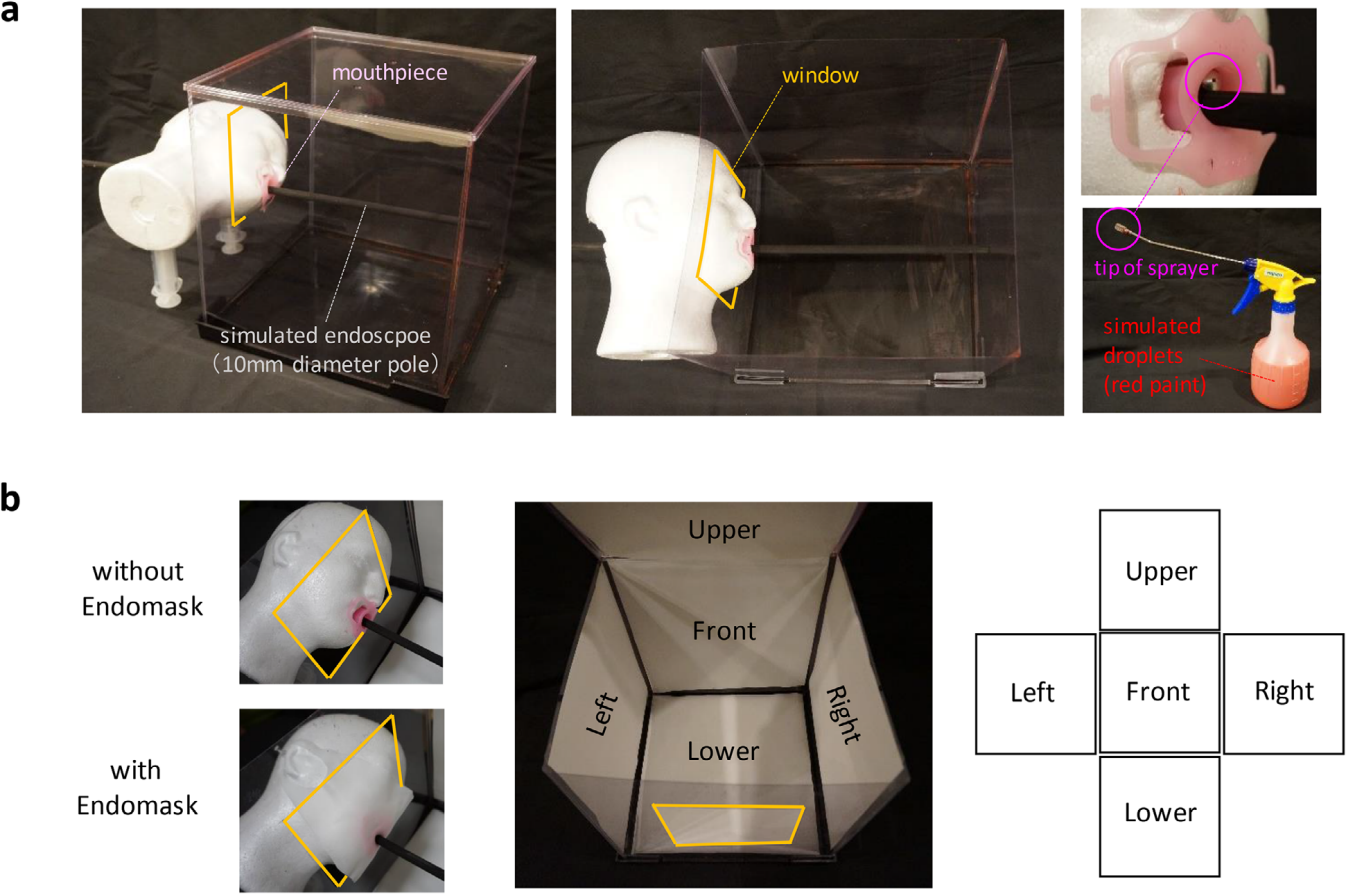
Experimental model that simulates the environment of EGD. (a) This experimental model consists of a mannequin head, mouthpiece, 10‐mm diameter pole simulating an endoscope and a 300‐mm plastic cube with a window. Simulated droplets of red paint were sprayed into the cube by a sprayer from 5 mm inside of the mouthpiece. (b) White papers were attached to the inner walls in advance in order to reveal the adherence of the paint. After spraying the simulated droplets, the white papers were peeled off and displayed like a cross. This experiment was repeated five times with or without Endomask

For quantitative evaluation of the efficacy, we established an experimental model that simulated the environment of EGD. A mannequin head was prepared and its mouth was hollowed out for fixing a mouthpiece and a 10 mm diameter pole simulating an endoscope. A 300 mm plastic cube with a window was placed in front of the mannequin head. Droplets from subjects were simulated by aqueous red paint. The paint was sprayed into the cube by a sprayer 10 times from 5 mm inside of the mouthpiece (Figure 2a). The constancy of the quantity of the sprayed ink was confirmed using a test tube in advance. The quantity of the paint ranged 6.5 ± 0.1 ml in the experiments repeated five times and was thought to be quite stable (data are not shown). White paper attached to the inner walls in advance could reveal the adherence of the paint. This experiment was repeated five times with or without Endomask (Figure 2b). The percentage of the area with paint was analyzed using computer software (Rasband, W.S., ImageJ, US National Institutes of Health, Bethesda, MD, USA).

### Safety

For evaluation of the safety, the saturation of oxygen and the expiratory carbonic dioxide partial pressure of subjects were measured by a pulse oximeter and capnometer (YG‐227T, Nihon Kohden Corporation, Tokyo, Japan) in 12 consecutive nonsedated subjects of EGD with Endomask. One of the subjects was diagnosed with chronic obstructive pulmonary disease. Each parameter was recorded four times (before EGD without Endomask, before EGD with Endomask, during EGD with Endomask, the end of EGD with Endomask). The record during EGD with Endomask was made when the scope passed through the pylorus. The values before EGD without our device served as the control.

### Stress on operability of endoscopists and respiration of subjects

Some questionnaires for the evaluation of stress on the operability of the endoscopists and the respiration of the subjects were prepared. Fifteen endoscopists (experts) were questioned after several examinations with Endomask. The questions were as follows: “Did you have any stress upon attaching this device?,” “Did you have any operation stress upon using this device?” They could answer for each question as follows: (i) no stress, (ii) a little stress (acceptable), (iii) moderate stress (unacceptable), (iv) serious stress. Ninety‐eight nonsedated subjects of EGD examined with Endomask were questioned on the respiratory stress and the understanding of the device. The questions were as follows: “Did you have any stress upon breathing with this device?,” “Did you understand the significance of this device?,” “Can you agree to the next EGD with this device?” They could answer the first question as follows: (i) no stress, (ii) a little stress (acceptable), (iii) moderate stress (unacceptable), (iv) serious stress. They answered the latter two questions as “Yes” or “No.”

### Statistical analysis

The parameters on the efficacy were analyzed by Mann–Whitney *U*‐test. The parameters on the safety were analyzed by paired *t*‐test. All analyses were done using Statcel 4 software (OMS Publishing Inc., Saitama, Japan).

## RESULTS

### Efficacy

**FIGURE 3:**
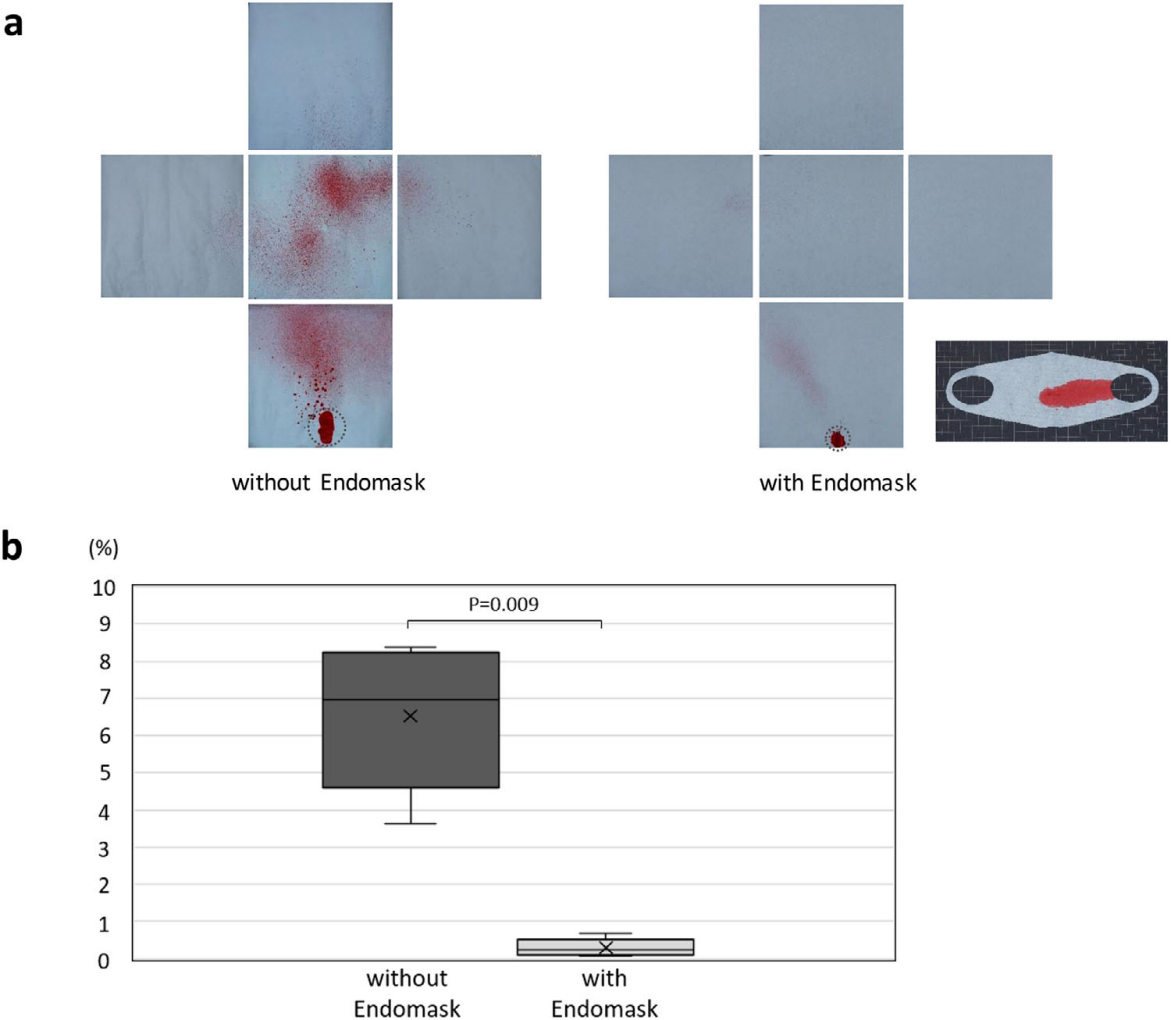
Results of the efficacy using the experimental model. (a) Endomask captured most of the simulated droplets and remarkably reduced the area with the paint. Circled paint adhered indirectly, dropping after the contact with the pole or Endomask. (b) Percentage of the area with the paint was significantly reduced by Endomask compared to that without Endomask

In the experimental model for quantitative evaluation of the efficacy, Endomask captured most of the simulated droplets and remarkably reduced the area with paint compared to that without the device (Figure 3a). In the experiments with Endomask, the percentage of the area with paint was significantly reduced compared to that without our device (median, 0.24% vs. 6.96%, *p* = 0.009) (Figure 3b).

### Safety

**FIGURE 4:**
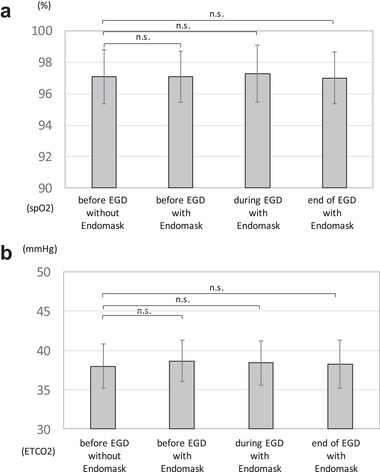
Results of the safety evaluated clinically. (a) Saturation of oxygen did not show any significant change at any recording time. (b) Expiratory carbonic dioxide partial pressure did not show any significant change at any recording time

The evaluation of the safety was conducted in 12 consecutive nonsedated subjects (11 males and a female) of EGD. Their median age was 75 years (range 56–90), and the median examination time was 232 s (range 182–317). In the clinical analysis for the evaluation of safety, the saturation of oxygen and the expiratory carbonic dioxide partial pressure of the subjects did not show any significant change at any recording times (Figure 4a,b).

### Stress on operability of endoscopists and respiration of subjects

The questionnaires for the endoscopists revealed that almost no endoscopists experienced stress when attaching this device and their operation stress was within acceptable level (Table 1). Ninety‐eight subjects (53 males and 45 females) of EGD were questioned; their median age was 63 years (range 29–90), and the median examination time was 291 s (range 182–754). The questionnaires for the subjects also revealed the minimal stress on breathing and complete understanding and acceptance of the device (Table 2).

**TABLE 1:** Questionnaires for the endoscopists

**Q1. Did you have any stress upon attaching this device?**	
No stress	14
A little stress (acceptable)	1
Moderate stress (unacceptable)	0
Serious stress	0
**Q2. Did you have any operation stress upon using this device?**	
No stress	11
A little stress (acceptable)	4
Moderate stress (unacceptable)	0
Serious stress	0

**TABLE 2:** Questionnaires for the subjects

**Q1. Did you have any stress upon breathing with this device?**	
No stress	79
A little stress (acceptable)	19
Moderate stress (unacceptable)	0
Serious stress	0
**Q2. Did you understand the significance of this device?**	
Yes	98
No	0
**Q3. Can you agree to the next EGD with this device?**	
Yes	98
No	0

## DISCUSSION

Among the reports on devices for the prevention of diffusion of droplets from subjects undergoing EGD, this is the first report that evaluates not only the efficacy but also the safety and the operability of the device to our knowledge.

We could show the ability of Endomask to prevent the diffusion of simulated droplets using an experimental model quantitatively. In this experimental model, Endomask significantly prevented the diffusion of simulated droplets. More than 95% of the simulated droplets were captured by Endomask. This result suggested that Endomask would effectively reduce the risk of contact and droplet transmission of SARS‐CoV‐2 during EGD. According to a recent report about the preventive effectiveness of face masks on airborne transmission of SARS‐CoV‐2,^23^ the protective efficiency was higher when masks were worn by a virus spreader. In that report, even cotton masks could block more than 50% of the virus and its potential was nearly equal to that of surgical masks when masks were worn by a virus spreader. Accordingly, Endomask is a reasonable solution and could be expected to partially reduce the risk of the airborne transmission of SARS‐CoV‐2, although an accurate evaluation of the diffusion of aerosols was difficult in this experimental model. In the report, a synergistic effect when both the virus receiver and virus spreader wore masks was also described. Therefore, the addition of Endomask to existing strategies including PPE of medical staffs could further reduce the risk of SARS‐CoV‐2 transmission.

We also showed the stability of respiration of subjects with Endomask during EGD. Our device changed neither the saturation of oxygen nor the expiratory carbonic dioxide partial pressure of the subjects at any recorded times. This suggested that Endomask could be used safely without obstructing the breathing of the subjects. Indeed, most subjects had no stress when breathing during EGD with Endomask according to the results of the questionnaire. The minimal stress of the device on breathing would promote acceptance by all subjects on the next use of the device. Before EGD, we explained the significance of the device to all the subjects. This process was thought to be important for understanding the purpose of the device.

With regard to the questionnaire for the endoscopists, the stress on both the attachment and the use of Endomask was within an acceptable level. This result suggested that Endomask was easy to use for the endoscopists. The minimal stress of the device for endoscopists is important given the expected frequent use.

The continuity of not only emergent but also elective endoscopic procedure should be considered even during the COVID‐19 pandemic, because postponement of an elective endoscopic procedure involves some risk of a delayed diagnosis of gastrointestinal diseases including malignancies. Lui et al. reported that a substantial reduction in endoscopic examinations during the COVID‐19 outbreak was associated with a significant delay in cancer diagnosis. They also mentioned that the delay and subsequent cancer stage upshifting will be amplified if COVID‐19 pandemic continues further.^24^ Under the continuation of the basic strategies (triage, appropriate PPE, careful management of subjects and the endoscopic room), Endomask could be useful as an additional solution against SARS‐CoV‐2 transmission during COVID‐19 pandemic.

The COVID‐19 pandemic necessitates the endoscopy staffs to reconsider the precautions against the infectious disease. Although the main precaution at the endoscopy units so far is the PPE, this would be insufficient for a pathogen with the risk of droplets/aerosol transmission like SARS‐CoV‐2. There are some other pathogens that also have the potential risk of droplets/aerosol transmission such as influenza,^25^ and such outbreaks cannot be predicted. Therefore, an additional standard precaution against the transmission of droplets/aerosols should be considered in endoscopy units. A simple and inexpensive device that can be utilized for all subjects is ideal as a standard precaution. Endomask could be a candidate as an additional standard precaution because it is easy to use and inexpensive.

A limitation of this study is that it was a single‐center pilot study and the number of participating subjects was small. In particular, although we demonstrated sustained respiratory parameters after using Endomask in this study, the number of subjects was too small for providing high evidence for safety of this device. Furthermore, the method of evaluating the stress of the endoscopists in this study may lack objectivity because it is a simple questionnaire survey at the developed facility. In the next step, a multicenter study, including the larger number of patients, with a more realistic survey (e.g., whether to continue to use the mask after its introduction) is warranted. Another limitation is that the efficacy of the device was evaluated not clinically but by using an experimental model. The constancy of speed of sprayed ink was not confirmed. Although accurate clinical evaluation of the ability to prevent the diffusion of droplets/aerosols is difficult, our experimental model would at least partially reflect the clinical condition of EGD.

In conclusion, Endomask could remarkably and safely reduce the diffusion of droplets without serious stress on the operators or the subjects of EGD. This inexpensive device could be a candidate as an additional standard precaution against some pathogens with the risk of droplets/aerosol transmission including SARS‐CoV‐2. Endomask is expected to reduce the infectious risk of SARS‐CoV‐2 in endoscopy units during the COVID‐19 pandemic.

## CONFLICT OF INTEREST

Hiroyuki Endo received the material of prototype from TOP Corporation. Author W.H. is an Associate Editor of *DEN Open*.

## ETHICS STATEMENT

This study was approved by the ethics committee of Japan Community Health Care Organization Sendai Hospital (#2020‐15) and was performed in accordance with the Declaration of Helsinki. All participants provided written informed consent before study participation.

## FUNDING INFORMATION

None.

## References

[1] Hussain A , Singhal T , El‐Hasani S . Extent of infectious SARS‐CoV‐2 aerosolisation as a result of oesophagogastroduodenoscopy or colonoscopy. Br J Hosp Med (London) 2020; 81: 1–7. 10.12968/hmed.2020.0348 32730160

[2] Sagami R , Nishikiori H , Sato T , *et al*. Aerosols produced by upper gastrointestinal endoscopy: A quantitative evaluation. Am J Gastroenterol 2021; 116: 202–5. 33079747 10.14309/ajg.0000000000000983

[3] Liu Y , Ning Z , Chen Yu , *et al*. Aerodynamic analysis of SARS‐CoV‐2 in two Wuhan hospitals. Nature 2020; 582: 557–60. 32340022 10.1038/s41586-020-2271-3

[4] Noorimotlagh Z , Jaafarzadeh N , Martínez SS , Mirzaee SA . A systematic review of possible airborne transmission of the COVID‐19 virus (SARS‐CoV‐2) in the indoor air environment. Environ Res 2021; 193: 110612. 33309820 10.1016/j.envres.2020.110612 PMC7726526

[5] Abd EW , Eassa SM , Metwally M , Al‐Hraishawi H , Omar SR . SARS‐CoV‐2 transmission channels: A review of the literature. MEDICC Rev 2020; 22: 51–69. 33295321 10.37757/MR2020.V22.N4.3

[6] Irisawa A , Furuta T , Matsumoto T , *et al*. Gastrointestinal endoscopy in the era of the acute pandemic of coronavirus disease 2019: Recommendations by Japan Gastroenterological Endoscopy Society (Issued on April 9th, 2020). Dig Endosc 2020; 32: 648–50. 32335946 10.1111/den.13703 PMC7267159

[7] Furuta T , Irisawa A , Matsumoto T , *et al*. Clinical questions and answers on gastrointestinal endoscopy during the novel coronavirus disease 2019 pandemic. Dig Endosc 2020; 32: 651–7. 32470171 10.1111/den.13757 PMC7301013

[8] He Xi , Lau EHY , Wu P , *et al*. Temporal dynamics in viral shedding and transmissibility of COVID‐19. Nat Med 2020; 26: 672–5. 32296168 10.1038/s41591-020-0869-5

[9] Ferretti L , Wymant C , Kendall M , *et al*. Quantifying SARS‐CoV‐2 transmission suggests epidemic control with digital contact tracing. Science 2020; 368: eabb6936. 32234805 10.1126/science.abb6936 PMC7164555

[10] Gao Z , Xu Y , Sun C , *et al*. A systematic review of asymptomatic infections with COVID‐19. J Microbiol Immunol Infect 2021; 54: 12–6. 32425996 10.1016/j.jmii.2020.05.001 PMC7227597

[11] Kronbichler A , Kresse D , Yoon S , Lee KH , Effenberger M , Shin JI . Asymptomatic patients as a source of COVID‐19 infections: A systematic review and meta‐analysis. Int J Infect Dis 2020; 98: 180–6. 32562846 10.1016/j.ijid.2020.06.052 PMC7832751

[12] Floriano I , Silvinato A , Bernardo WM , Reis JC , Soledade G . Accuracy of the polymerase chain reaction (PCR) test in the diagnosis of acute respiratory syndrome due to coronavirus: A systematic review and meta‐analysis. Rev Assoc Med Bras 2020; 66: 880–8. 32844930 10.1590/1806-9282.66.7.880

[13] De Grazia F , Marconi S , Bardone M , *et al*. Use of 3D printer for face mask production to protect endoscopy unit personnel in contact with high‐risk patients during COVID‐19 pandemic. Endoscopy 2020; 52: 1146–7. 33105505 10.1055/a-1206-0937 PMC7724583

[14] Lazaridis N , Skamnelos A , Murino A , Chacchi Cahuin R , Koukias N , Despott EJ . “Double‐surgical‐mask‐with‐slit” method: Reducing exposure to aerosol generation at upper gastrointestinal endoscopy during the COVID‐19 pandemic. Endoscopy 2020; 52: 928–9. 32967022 10.1055/a-1198-5471 PMC7516391

[15] Maruyama H , Higashimori A , Yamamoto K , *et al*. Coronavirus disease outbreak: A simple infection prevention measure using a surgical mask during endoscopy. Endoscopy 2020; 52: E461–2. 32818997 10.1055/a-1220-6024 PMC7724578

[16] Marchese M , Capannolo A , Lombardi L , Di Carlo M , Marinangeli F , Fusco P . Use of a modified ventilation mask to avoid aerosolizing spread of droplets for short endoscopic procedures during coronavirus COVID‐19 outbreak. Gastrointest Endosc 2020; 92: 439–40. 32247754 10.1016/j.gie.2020.03.3853 PMC7194597

[17] Bojórquez A , Larequi FJZ , Betés MT , Súbtil JC , Muñoz‐Navas M . Commercially available endoscopy facemasks to prevent aerosolizing spread of droplets during COVID‐19 outbreak. Endosc Int Open 2020; 8: E815–6. 32529025 10.1055/a-1180-8355 PMC7280021

[18] Gomi K , Nagahama M , Yoshida E , Takano Y , Kuroki Y , Yamamoto Y . Peroral endoscopy during the COVID‐19 pandemic: Efficacy of the acrylic box (Endo‐Splash Protective (ESP) box) for preventing droplet transmission. JGH Open 2020; 4: 1224–8. 33319060 10.1002/jgh3.12438 PMC7731832

[19] Campos S , Carreira C , Marques PP , Endoprotector VA . Protective box for safe endoscopy use during COVID‐19 outbreak. Endosc Int Open 2020; 8: E817–21. 32509960 10.1055/a-1180-8527 PMC7266658

[20] Kobara H , Nishiyama N , Masaki T . Shielding for patients using a single‐use vinyl‐box under continuous aerosol suction to minimize SARS‐CoV‐2 transmission during emergency endoscopy. Dig Endosc 2020; 32: e114–5. 32492223 10.1111/den.13713 PMC7300621

[21] Sagami R , Nishikiori H , Sato T , Murakami K . Endoscopic shield: Barrier enclosure during the endoscopy to prevent aerosol droplets during the COVID‐19 pandemic. VideoGIE 2020; 5: 445–8. 32395674 10.1016/j.vgie.2020.05.002 PMC7211574

[22] Endo H , Koike T , Masamune A . Novel device for preventing diffusion of aerosol droplets from subjects undergoing esophagogastroduodenoscopy during COVID‐19 pandemic. Dig Endosc 2020; 32: e140–1. 32696996 10.1111/den.13772 PMC7405108

[23] Ueki H , Furusawa Y , Iwatsuki‐Horimoto K , *et al*. Effectiveness of face masks in preventing airborne transmission of SARS‐CoV‐2. mSphere 2020; 5: e00637–20. 33087517 10.1128/mSphere.00637-20 PMC7580955

[24] Lui TKL , Leung K , Guo C‐G , Tsui VWM , Wu JT , Leung WK . Impacts of the coronavirus 2019 pandemic on gastrointestinal endoscopy volume and diagnosis of gastric and colorectal cancers: A population‐based study. Gastroenterology 2020; 159: 1164–66. 32425228 10.1053/j.gastro.2020.05.037 PMC7230139

[25] Asadi S , Gaaloul Ben Hnia N , Barre RS , Wexler AS , Ristenpart WD , Bouvier NM . Influenza A virus is transmissible via aerosolized fomites. Nat Commun 2020; 11: 4062. 32811826 10.1038/s41467-020-17888-w PMC7435178

